# Using remotely sensed night-time light as a proxy for poverty in Africa

**DOI:** 10.1186/1478-7954-6-5

**Published:** 2008-10-21

**Authors:** Abdisalan M Noor, Victor A Alegana, Peter W Gething, Andrew J Tatem, Robert W Snow

**Affiliations:** 1 Malaria Public Health and Epidemiology Group, Centre for Geographic Medicine, KEMRI – University of Oxford – Wellcome Trust Collaborative Programme, Kenyatta National Hospital Grounds (behind NASCOP), P.O. Box 43640-00100, Nairobi, Kenya; 2 Centre for Tropical Medicine, John Radcliffe Hospital, University of Oxford, Oxford, OX3 9DS, UK; 3 Centre for Geographic Health Research, School of Geography, University of Southampton, Southampton, SO17 1BJ, UK; 4 Spatial Ecology and Epidemiology Group, Tinbergen Building, Department of Zoology, University of Oxford, South Parks Road, Oxford, OX1 3PS, UK

## Abstract

**Background:**

Population health is linked closely to poverty. To assess the effectiveness of health interventions it is critical to monitor the spatial and temporal changes in the health indicators of populations and outcomes across varying levels of poverty. Existing measures of poverty based on income, consumption or assets are difficult to compare across geographic settings and are expensive to construct. Remotely sensed data on artificial night time lights (NTL) have been shown to correlate with gross domestic product in developed countries.

**Methods:**

Using national household survey data, principal component analysis was used to compute asset-based poverty indices from aggregated household asset variables at the Administrative 1 level (n = 338) in 37 countries in Africa. Using geographical information systems, mean brightness of and distance to NTL pixels and proportion of area covered by NTL were computed for each Administrative1 polygon. Correlations and agreement of asset-based indices and the three NTL metrics were then examined in both continuous and ordinal forms.

**Results:**

At the Administrative 1 level all the NTL metrics distinguished between the most poor and least poor quintiles with greater precision compared to intermediate quintiles. The mean brightness of NTL, however, had the highest correlation coefficient with the asset-based wealth index in continuous (Pearson correlation = 0.64, p < 0.01) and ordinal (Spearman correlation = 0.79, p < 0.01; Kappa = 0.64) forms.

**Conclusion:**

Metrics of the brightness of NTL data offer a robust and inexpensive alternative to asset-based poverty indices derived from survey data at the Administrative 1 level in Africa. These could be used to explore economic inequity in health outcomes and access to health interventions at sub-national levels where household assets data are not available at the required resolution.

## Background

The health of populations is inextricably linked to the depth of their poverty [1,2]. Breaking the vicious cycle of poverty and ill-health has formed the basis of the international community's Millennium Development Goals (MDGs) [3]. At national levels, targeting resources to those most in need is a guiding principle of poverty reduction strategies and health policies [4]. However, obtaining accurate metrics on the depth and spatial disparities in poverty poses several problems. Measures of poverty at household level are often computed from complex survey data on income, consumption or expenditure [5]. These data are difficult to reliably collect at regular intervals nationally; are subject to significant reporting bias; show large fluctuations over time; or are seen as indicative only of the short term economic status of the sampled households [6,7]. A default metric that is used more frequently, and is easier to collect during household surveys, is based on assets variables [6,8]. In sub-Saharan Africa (SSA), most national household surveys now have a standardized welfare module that routinely collects information on household assets and are used to report the socio-economic patterns in health outcomes [9,10]. Several of these common asset variables have also been shown to be associated with income and consumption [11,12] and this relationship is now the basis of poverty mapping using small-area estimation methods [13,14].

Asset-based wealth indicators, although easier to collect, suffer from limitations similar to those of income- and consumption-based indicators often resulting in metrics that are not comparable across countries, or even within countries, especially where the relationship of input variables to well-being varies across different social and geographical settings [6,15]. Therefore, where the aim is to relate poverty to other metrics such as health across multiple geographic entities, these poverty measures become deficient. Furthermore, regardless of which survey-based measure of poverty is used, the process of collecting the relevant data to allow the examination of detailed sub-national differences in poverty and resource need is expensive. Alternative measures are therefore required that are easier to interpret, comparable temporally and spatially across national and sub-national boundaries and for which data are less expensive to obtain.

The spatial distribution and intensity of satellite-derived night time lights (NTL) has been shown in several studies to correlate with per capita gross domestic product (GDP) and other national level socio-economic indicators [15-19]. It has also been shown to be a good proxy for population distribution [20]. This simple source of information is derived from satellite imagery at high spatial resolutions and is readily available in the public domain [18]. However, until now analysis using NTL as a proxy for poverty has only considered its relationship with consumption-based measures in high-income countries [15,19] where such data exist. Here we seek to examine the correlation between NTL and wealth asset indicators of poverty at sub-national spatial resolutions in Africa.

## Methods

### Data

#### Units of analysis: Administrative 1 unit

The Administrative 1 unit, which is the equivalent of provinces, states or regions in most African countries and considered to be the second tier of government after the national level [21], was used as the spatial unit of analysis. Digital maps of these units were obtained through a combination of the United Nations Geographic Information Working Group – Second Administrative Level Boundary (UNGIWG-SALB) and the Food & Agriculture Organization – Global Administrative Units Layers (FAO-GAUL). The UNGIWG-SALB project began in the mid-1990s as an effort to develop agreed-upon digital boundaries to at least the second administrative level for purposes of developing a global population grid surface [22]. This attempt was based on a standardized international borders template developed by the UN Cartographic Section involving an elaborate network of UN and other agencies and national governments [21]. The FAO-GAUL initiative is funded by the European Commission (EC) and works along similar structures as the UNGIWG-SALB effort [23]. However, there were differences in the resolution of the two boundary datasets and they were therefore combined and the data with finest resolution was retained to create a comprehensive digital boundary database at Administrative 1 level [24].

#### Zero population mask

**Figure 1:**
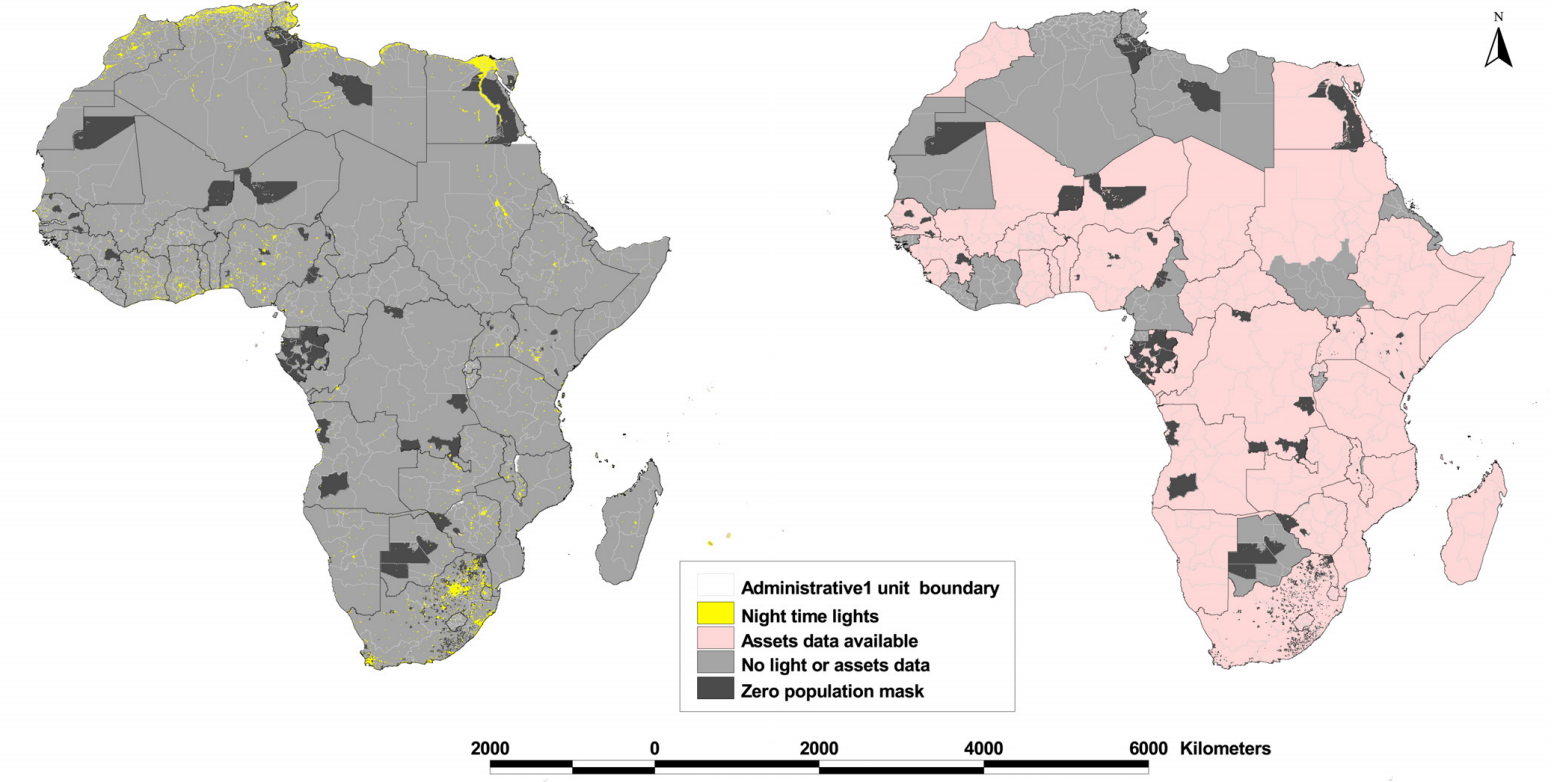
**Administrative 1 unit boundary maps of Africa showing: a) the distribution of night time lights for the year 2000; b) availability of assets data for 338 units in 37 countries.** The maps also show areas of zero population density derived from GRUMP surface.

The Global Rural Urban Mapping Project (GRUMP) is the most recent and highest resolution source of human population distribution data at the continental level [25]. This database is created from a substantially larger number of administrative data units, and has been shown to provide a higher level of accuracy, than other population data products [26,27]. GRUMP provides global gridded population density estimates at ~1 × 1 km spatial resolution as described in detail elsewhere [25,28]. Those areas of Africa defined by GRUMP as having zero population were vectorized to form polygons (Figure 1) which were then used to re-define the habitable area within each Administrative 1 unit for subsequent extraction and analysis.

#### Extraction of NTL data

The Defense Meteorological Satellite Program (DMSP) Operational Linescan System (OLS) instruments measure emitted visible and infrared radiation and at night time produce imagery of lights on the ground (NTL imagery). By compositing cloud-free NTL images and reporting the frequency of observations above a threshold average radiance, global NTL products can be produced. Moreover, by removing ephemeral lights produced by fires and random noise events that occurred in the same place less than three different times, 'stable' lights can be identified. These stable lights represent electrified human settlements, gas flares and heavily lit boats, primarily. Based on location, brightness, persistence and visual appearance, these are separated into separate global products [30]. The global human settlement NTL product at ~1 × 1 km spatial resolution for the year 2000 was downloaded from the the National Oceanic and Atmospheric Administration's National Geophysical Data Center (NOAA-NGDC) website [31] in raster grid format and data for Africa were extracted (Figure 1). The brightness of light pixels vary on an arbitrary scale from 0–63 units, which represents the average brightness for 2000, with the centre of large, well-electrified cities producing the highest values. The total habitable area under NTL, defined as anywhere with a brightness value of 1 or greater, was computed for each Administrative 1 unit using ArcGIS 9.1 (ESRI Inc., NY, USA) extraction tools. In addition, the mean of brightness of and great circle distances (km) to light pixels were computed for each Administrative 1 unit. Administrative 1 units were then ranked into quintiles using these extracted light pixel parameters.

#### Household assets information

Most standard national surveys in the last decade have captured information on a variety of household level asset variables: household head education and occupation; household ownership of durable goods; access to water and sanitation; and type of housing structure which are used as proxies of household wealth. The two main sources of household assets data used in this study were the Multiple Indicators Cluster Surveys (MICS) supported by the United Nations Children's Fund (UNICEF) [9] and the Demographic and Health Surveys (DHS) implemented and managed by MEASURE (Monitoring and Evaluation to Assess and Use Results) – DHS [10] in collaboration with national ministries and statistics bureaus. UNICEF developed MICS methodologies in the mid-1990s and began the first round (MICS 1) in 1995 followed by a second round (MICS 2) in 2000 covering a total of 24 African countries [9]. The third and most recent round (MICS 3) was undertaken from 2005–2007 and covered 19 countries in Africa [32]. Both DHS and MICS are designed to be representative at the national and Administrative 1 level with generally large sample sizes of approximately 5,000 households or more derived from a two-stage cluster sample design and are usually conducted every five years.

**Figure 2:**
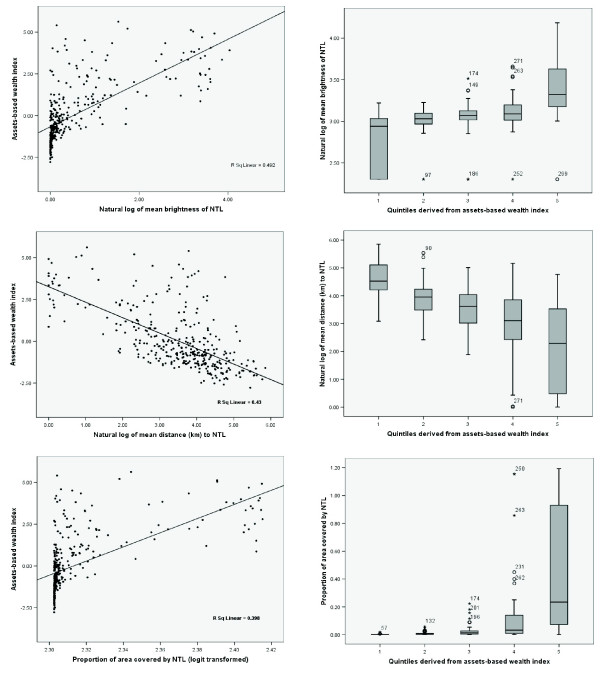
Scatter and box* plots showing the relationship of the asset index against mean** brightness of NTL; mean distance to NTL; and proportion of area covered by NTL. The *x*-axis of the box plots show quintiles derived from the asset-based index where 1 = most poor and 5 = least poor. *The box indicates the inter-quartile range (25% and 75%) and the thick line within the box represents the median. The whiskers represent the 2.5% and 97.5% percentiles and outliers are plotted as circles outside this range. **The mean includes pixels with zero nigh time pixel values.

Several countries have multiple MICS and DHS data available in the public domain, but for the purpose of this analysis, priority was given to household surveys that were undertaken close to the year 2000, the year of production of the NTL data (Table 1). Selected surveys for all countries were then compared in terms of the types and categories of household level asset variables that they contained. Only those variables that were common across all countries were selected, including: household head education (no education, primary, secondary & above); ownership of durable goods (radio and television); access to piped water; and connection to sewage system (Table 1). Of 56 African countries, only 37 had comparable national household surveys available, 11 of which were carried out between the years 2003 and 2006 (Figure 2 & Table 1). It was decided that these surveys carried out between these years were sufficiently close in time to the 2000 NTL data for meaningful comparison given that asset indicators are less volatile and not subject to fluctuations in the short term compared to the standard income and consumption measures [11].

**Table 1:** Country (n = 37) summaries.

				**Assets-based wealth index**	**Brightness of night time lights (NTL)**	**Distance (km) to NTL**	**Distance to nearest BTL pixel**	
**Country**	**Source**	**Survey Year**	**Number of Admin1 units (n = 338)**	**Mean (standard deviation)**	**Mean (standard deviation)**	**Mean (standard deviation)**	**Mean (standard deviation)**	**% area covered by NTL**

Angola	MICS	2001	18	-1.02 (0.80)	0.0357 (0.9587)	89.98 (50.77)	71.86(36.23)	0.31

Benin	DHS	2001	6	-0.29 (0.86)	0.2398 (2.1562)	38.43 (26.68)	25.41(16.50)	2.16

Burkina Faso	DHS	2003	13	-0.97 (0.69)	0.1292 (1.7804)	40.28 (29.25)	32.90(17.24)	0.99

CAR	DHS	1994–5	17	-1.54 (0.82)	0.0097 (0.4832)	213.89 (111.01)	163.71(44.66)	0.10

Chad	DHS	2004	9	-1.13 (1.07)	0.0061 (0.3419)	220.48(114.45)	133.85(47.42)	0.07

Comoros	DHS	1996	3	-0.48 (0.20)	0.8398 (2.4499)	6.59(5.89)	7.07(5.31)	12.29

Congo	DHS	2005	4	1.38 (1.57)	0.0393 (1.0331)	82.44(40.53)	57.02(20.28)	0.31

DRC	MICS	2001	11	-0.75 (0.93)	0.0287 (0.8941)	152.98(94.61)	123.63(65.11)	0.23

Egypt	DHS	2000	26	3.45 (1.16)	1.9321 (8.1347)	76.02(74.41)	16.37(13.99)	12.18

Ethiopia	DHS	2000	11	-0.89 (1.38)	0.0541 (0.9639)	70.8(44.60)	53.85(29.15)	0.54

Gabon	DHS	2000	5	1.23 (1.59)	0.0786 (1.4102)	52.26(32.34)	49.32(31.26)	4.02

Gambia	MICS	2000	6	0.9 (1.49)	0.3702 (2.5203)	16.42(9.94)	13.97(8.04)	3.76

Ghana	DHS	2003	10	0.62 (1.41)	0.8348 (4.1937)	17.13(14.66)	13.92(10.23)	7.65

Guinea	DHS	2005	8	-0.70 (1.20)	0.1335 (1.5688)	36.51(22.37)	31.89(17.33)	1.30

Kenya	DHS	2003	8	0.20 (1.75)	0.1775 (1.8377)	63.22(41.51)	38.64(23.23)	1.72

Lesotho	MICS	2000	10	-0.12 (0.56)	0.4178 (2.8363)	23.08(15.62)	22.05(10.27)	3.58

Madagascar	DHS	2003–4	6	-1.06 (1.09)	0.0393 (0.8856)	84.13(47.13)	74.18(38.23)	0.36

Malawi	DHS	2000	3	-0.73 (0.05)	0.5109 (3.1261)	20.94(15.55)	20.66(13.18)	4.83

Mali	DHS	2001	7	-0.37 (1.43)	0.0271 (0.7588)	188.99(165.15)	78.45(49.63)	0.24

Morocco	DHS	2003–4	7	2.89 (0.55)	1.0878 (5.0683)	18.59(16.89)	15.73(12.61)	8.79

Mozambique	DHS	2003	11	-0.64 (1.03)	0.0624 (1.2019)	71.55(43.71)	62.02(33.21)	0.54

Namibia	DHS	2000	13	1.73 (1.91)	0.0799 (1.2678)	61.71(43.14)	55.30(33.97)	0.75

Niger	DHS	1998	8	-1.11 (0.91)	0.0203 (06527)	155.43(95.34)	94.36(53.57)	0.22

Nigeria	DHS	2003	6	0.39 (0.74)	0.5254 (3.2639)	27.94(26.51)	25.30(21.27)	4.54

Rwanda	MICS	2000	12	-1.00 (0.83)	0.3252 (2.7895)	25.26(16.20)	21.14(8.37)	2.53

Sao Tome and Principe	MICS	2000	2	0.78 (0.18)	1.1627 (4.2087)	34.11(51.85)	84.42(7.50)	10.02

Senegal	MICS	2000	10	0.46 (1.21)	0.2337 (2.2721)	34.77(25.89)	21.26(13.28)	2.49

Sierra Leone	MICS	2000	4	0.58 (1.13)	0.0349 (0.6409)	40.03(19.65)	31.78(15.43)	0.43

Somalia	MICS	2006	18	-1.67 (0.88)	0.0097 (0.3541)	112.97(59.56)	104.62(39.20)	0.10

South Africa	DHS	1998	9	2.20 (1.19)	1.6346 (6.4894)	22.42(42.23)	15.87(23.57)	13.45

Sudan	MICS	2000	16	-0.50 (0.78)	0.0774 (1.311)	129.60(104.62)	83.93(52.13)	0.87

Swaziland	MICS	2000	4	1.75 (0.67)	1.8261 (5.3554)	7.56(6.28)	7.55(5.91)	17.28

Tanzania	DHS	1999	9	-0.73 (0.87)	0.096 (1.3980)	59.90(38.57)	46.27(28.37)	0.86

Togo	MICS	2000	5	-0.16 (0.20)	0.428 (2.9724)	22.85(15.63)	22.45(13.81)	3.70

Uganda	DHS	2000–1	4	-0.74 (0.68)	0.1649 (1.9448)	46.21(28.16)	43.77(24.73)	1.39

Zambia	DHS	2001–2	9	-0.06 (1.55)	0.1684 (1.9745)	49.93(28.42)	46.34(26.96)	1.30

Zimbabwe	DHS	1999	10	1.06 (2.55)	0.4490 (3.2131)	32.09(22.92)	23.92(15.74)	4.17

Showing type and year of national surveys used to constructing asset indices: the mean (standard deviation) of asset indices; brightness of night time lights; distance to the nearest light pixel; and the percentage of area covered by NTL at the Administrative1 level units.

CAR = Central African Republic

DRC = Democratic Republic of Congo (formerly Zaire)

DHS = Demographic and Health Surveys

MICS = Multiple Indicator Cluster Surveys

#### Constructing wealth assets index at administrative1 level

The five selected household level assets variables were aggregated to Administrative 1 level digital boundaries in ArcGIS 9.1 by calculating the proportion of households in each response category (Table 1). A wealth assets index was then computed for each Administrative 1 unit from these aggregated assets variables using principal component analysis (PCA). PCA is a data reduction technique that provides a method of identifying, from a multivariate data set, weighted combinations of variables that contain most of the information common to the full set [8]. The first principle component represented the linear combination of asset variables which explained the largest proportion of the total variation in the data set, and was used to represent a composite wealth assets measure. The corresponding component loading weights quantified the contribution of each variable to this composite measure. These loadings were then used to compute a weighted sum of the proportions in each Administrative 1 unit to create a single composite wealth assets index that encapsulated most of the information contained in the categories of the five separate assets variables. Values of this index were then used to rank Administrative 1 units into quintiles.

#### Assets index versus night-time lights as a measure of poverty

**Figure 3:**
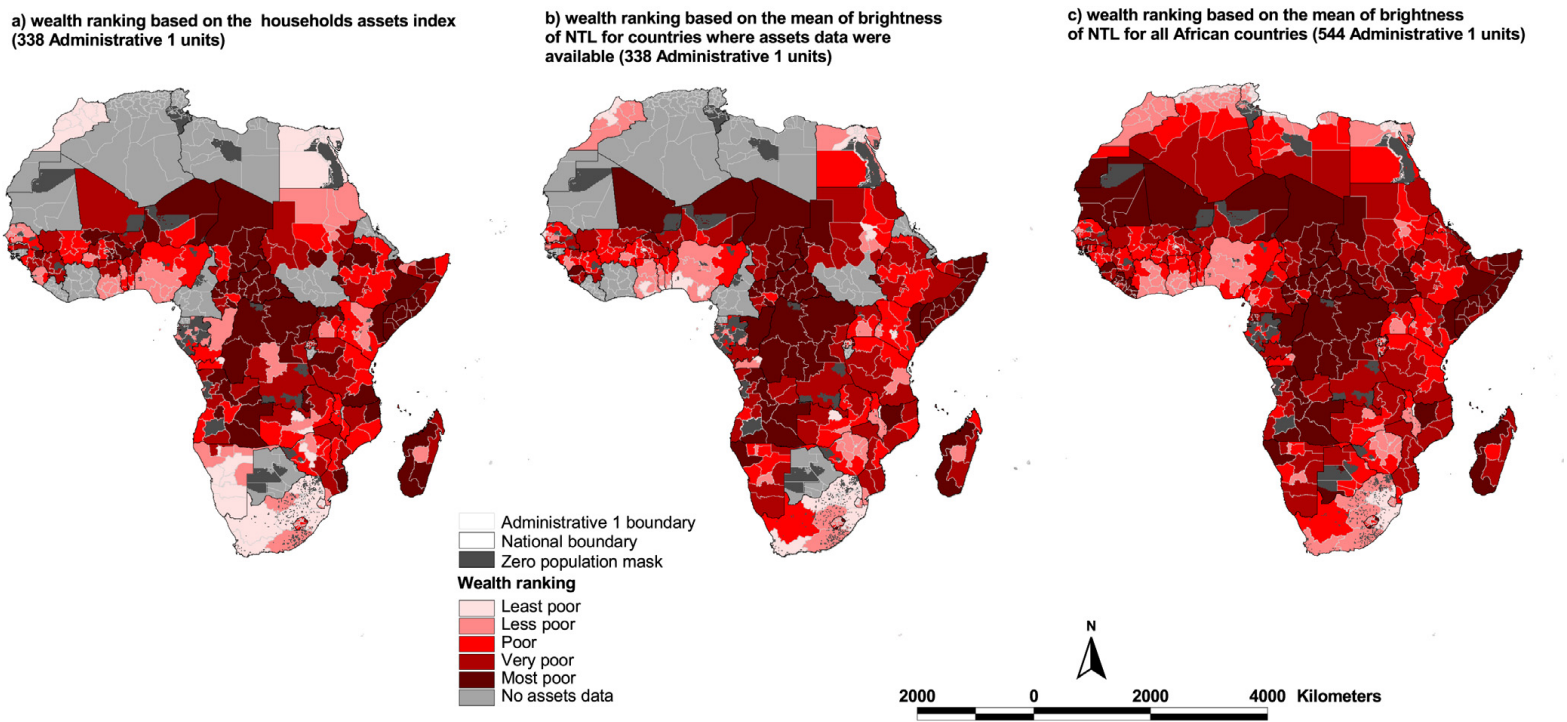
Administrative 1 units boundary maps of Africa comparing wealth rankings based on the asset index and those based on the mean brightness of night time lights.

An Administrative 1 level comparison between wealth assets index and both the mean brightness of and distance to nearest NTL pixel and the proportion of area covered by NTL was undertaken using scatter plots and Pearson's correlation tests. The variables were all transformed using natural logarithms and were then examined visually for normality. A constant of value one was added to the NTL metrics before transformation to account for those Administrative 1 units with original values of zero. In addition, box-plots of the NTL measures categorized by the assets-based wealth quintiles were constructed. The relationships between quintile rankings of Administrative 1 units based on the asset index and on all three NTL metrics were investigated using the Spearman's rank correlation and Kappa statistics. The Kappa statistic ranges from 0 to 1 with values < 0.01 indicating less than chance agreement; 0.01–0.20 slight agreement; 0.21–0.40 fair agreement; 0.41–0.60 moderate agreement; 0.61–0.80 substantial agreement; and 0.81–0.99 almost perfect agreement [33]. Maps of Administrative 1 units showing the ranking of units based on the asset index and the NTL metric with the highest correlation were generated in ArcGIS 9. (Figure 3).

## Results

Comparable household assets data were available for 338 Administrative 1 units in 37 out of 56 African countries (Figure 1 & Table 1). Assets data for 15 out of 37 countries were obtained from surveys done in the year 2000 (corresponding to the production year of the NTL): seven countries in 1999 or 2001; two countries in 1998; and the rest of the countries in 2003 – 2006 (Table 1). The first component from which the asset index was derived explained 43.3% of the variation in the asset data. Overall the wealth index based on the five asset variables ranged from a mean of -1.67 in Somalia to 3.45 in Egypt. The mean (standard deviation) of brightness of light pixels ranged from 0.0061 (0.3419) digital numbers in Chad to 1.9321 (8.1347) in Egypt. Chad and Somalia ranked as the countries with lowest mean brightness of NTL each with a value of 0.0097. Overall, 2.2% of the total area of the 37 countries was covered by NTL, ranging from 0.07% in Chad to 17.28% in Swaziland while Egypt had 12.18% of area covered by NTL. The mean distance to nearest NTL pixel was highest for Central African Republic (163.71 km) and lowest for Comoros Islands (7.07 km). Overall, 26 out of 338 Administrative 1 units did not have any NTL pixels.

According to the asset index 18 out of 37 countries did not have a single Administrative 1 unit in the least poor quintile, with 97 out of 165 units in these countries ranked in the poorest and second poorest quintiles (Table 2& Figure 3). Among those 18 countries which did not have Administrative 1 units in the least poor quintile, Somalia, Chad, Central African Republic, Niger and Angola had 50% or more of their units in the most poor quintile. In contrast, all of 7 and 26 Administrative 1 units in Morocco and Egypt respectively were in the least poor quintile. When the quintile rankings based on the mean brightness and distance to, and proportion of area covered by, NTL were considered, the countries that dominated the bottom and top quintiles generally remained the same with Chad, Somalia and the Central African Republic consistently ranked as the 'poorest' while Egypt, Morocco, Swaziland and South Africa the 'richest' (Table 2).

**Table 2:** Ordinal wealth rankings (quintiles) of 338 Administrative 1 units in 37 African countries.

	**Assets- based wealth index**					**Mean brightness of night time lights (NTL)**					**Proportion of area covered by NTL**					**Mean distance (km) to the nearest NTL**				
**Country**	**1**	**2**	**3**	**4**	**5**	**1**	**2**	**3**	**4**	**5**	**1**	**2**	**3**	**4**	**5**	**1**	**2**	**3**	**4**	**5**

Angola	6	6	4	2		5	11	1		1	5	10	2		1	6	9	2		1

Benin		3	1	2				2	2	2			2	2	2			3		3

Burkina Faso	5	3	4	1		1	3	7	1	1		4	8		1		2	2	8	1

CAR	13	2	1	1		13	2	1		1	13	2	1		1	12	3	1		1

Chad	5	1	2	1		5	3			1	5	3			1	6	1	1		1

Comoros			3					1	2					2	1					3

Congo			1	1	2	2	1			1	2	1			1	1	2			1

DRC	2	6	1	1	1	7	2		1	1	7	2		2		9	1	1		

Egypt					26			1	4	21			2	3	21	1	2	3	3	17

Ethiopia	4	1	4	1	1	3	4	2		2	5	2	2		2	2	5	1	1	2

Gabon			1	3	1		2	2		1		2	2		1		2	3		

Gambia			1	4	1		1	3	1	1			3	2	1				5	1

Ghana		3		6	1			1	6	3			1	5	4			1	4	5

Guinea	1	5	1		1		2	4	1	1		2	4	1	1		1	4	2	1

Kenya	1		4	2	1	1		3	2	2	1		3	2	2	1	3		2	2

Lesotho		1	5	4		2		2	6		1	1	2	6				2	6	2

Madagascar	3	2		1		2	2	1	1		2	2	2			2	2	2		

Malawi			3					1	2				1	2				1	2	

Mali	1	2	3		1	1	3	2		1	1	3	2		1	2	2	2		1

Morocco					7				5	2				5	2				4	3

Mozambique	2	4	3	1	1	1	5	3	1	1	1	6	2	1	1	3	5	1	1	1

Namibia		2		5	6	1	3	7	2		2	2	6	3		1	7	4	1	

Niger	4	2	1	1		2	3	2		1	2	4	1		1	4	2	1		1

Nigeria			2	4				1	3	2			1	4	1			2	4	

Rwanda	3	7	1	1		4	1	2	4	1	4		2	5	1			3	6	3

Sao Tome and Principe				2		1				1	1				1	1			1	

Senegal		1	3	5	1		1	3	4	2		1	3	4	2			3	4	3

Sierra Leone			1	2	1	1	2			1	1	2			1			3		1

Somalia	13	2	1	2		14	2	1		1	13	4			1	12	4	1		1

South Africa				2	7				3	6			1	2	6			1	5	3

Sudan	1	8	2	5		2	7	3	2	2	2	7	3	2	2	5	7	3	1	

Swaziland				1	3				2	2				1	3					4

Tanzania	2	3	3	1			4	2	2	1		4	2	2	1		4	3		2

Togo			3	2				1	3	1			1	3	1			1	3	1

Uganda	1	1	1	1			1	2	1			1	2	1			1	3		

Zambia	1	2	3	1	2		3	4	1	1		3	4	2			3	6		

Zimbabwe		1	5	1	3			3	5	2			3	5	2			4	4	2

Based on household assets-based indices; the mean brightness of night time lights; the mean distance to nearest night time lights pixel; and the proportion of area covered by night time lights. Q1 = most poor quintile; Q5 = least poor quintile.

Scatter and box plots of the continuous and ordinal (quintile) relationships between the asset-based wealth index and the three NTL measures at Administrative 1 level are shown in Figure 2. While mean brightness of, and proportion of area covered by, NTL exhibited positive correlation with the assets-based index, the mean distance to nearest NTL pixel, as anticipated, showed a negative correlation. All the NTL indicators distinguished unambiguously between the most and least poor quintiles based on the assets index. Their strength, however, in separating the middle quintiles was generally weak (Figure 2). The Pearson and Spearman correlation coefficients of the asset index versus the three NTL measures are presented in Table 3. In the continuous form, the mean brightness of NTL exhibited the strongest correlation with asset-based wealth index of all three NTL indicators (Pearson correlation = 0.64, p < 0.01) (Table 3). When the quintiles based on the assets-based wealth index were compared to those based on the three NTL measures, the quintile rankings of the mean of NTL brightness had the highest Spearman's rank correlations of 0.79 while those of the mean distance to nearest NTL pixel had the lowest correlation (-0.62) with the asset-based index. The corresponding Kappa statistic was 0.64 and 0.58 showing substantial and moderate agreement with assets index respectively (Table 3).

**Table 3:** Pearson correlation; Spearman rank correlation; and Kappa statistics of the relationship between the asset-based wealth index and the various night time lights metrics for 338 Administrative 1 level units in 37 countries in Africa.

	Pearson* Correlation	Spearman's* rank correlation	**Kappa coefficient (95% CI)**
Mean brightness of NTL	0.64	0.79	0.64 (0.70, 0.58)

Proportion of area covered by NTL	0.63	0.74	0.58 (0.63, 0.51)

Mean distance (km) to NTL	-0.61	-0.62	0.42 (0.49, 0.35)

The Pearson and Spearman's correlations assess the relationships between the asset-based wealth index and the night time lights metrics in the continuous and the categorical (quintiles) forms, respectively. CI = confidence interval

*Correlations are significant at the 0.01 level (2-tailed)

## Discussion

Currently international development milestones such as the MDGs, which comprise a set of eight internationally agreed goals that cover areas such as poverty reduction, education, infrastructure and health, use asset-based wealth quintiles as a way of monitoring changes in socio-economic inequity [34]. NTL, whilst representing a narrower dimension of human development compared to the combined asset variables of wealth, provide the benefit of being easily available and comparable spatially and temporally at a high spatial resolution. In this study we have shown that the mean brightness of the NTL human settlement product had a reasonably high linear correlation with asset-based indices at the Administrative 1 unit level in Africa (Table 3 & Figure 2) as both a continuous (Pearson's correlation coefficient = 0.64) and ordinal (Spearman's correlation coefficient = 0.79; Kappa = 0.64) variable. The ordinal forms of all the NTL metrics clearly separated the most and least poor quintiles with the median asset-based index of these quintiles not overlapping (Figure 2). While we have examined solely the use of 2000 NTL data here, the forthcoming production of more contemporary human settlement products [31], the constant acquisition of new NTL imagery [35] and even the possibility of finer resolution NTL imagery [36] mean that the potential to track changes in poverty levels over large scales exists, and this will be a focus of future research.

The main attraction of presenting poverty or socio-economic data on an ordinal scale, such as quintiles, is the ease with which results can be interpreted by policy makers and planners. This is especially the case when such a scale is used to define heterogeneity in specific population indicators such as fertility, mortality or access to public services. The problem with ordinal scales, however, is that information in intermediate classes, (2nd, 3^rd ^and 4^th ^in the case of quintiles), is rarely distinct and difficult to interpret. Consequently, most studies and programmes focus mainly on the difference between the top (least poor) and bottom (most poor) quintiles. In this regard, the significant positive correlation between asset indices and the mean brightness of NTL, particularly in the ordinal form, provides an opportunity for using the latter as an alternative poverty metric to asset-based indices, with the additional benefit of preserving independence and comparability across geographic settings, particularly in most of Africa where the use of electric lighting remains generally low with significant between and within country variation [37]. Arguably, as more recent national survey data that record household level variables become available, the need for such NTL metrics will decrease for within country evaluations. In addition, it is possible the NTL metric is a weak proxy of poverty at cluster level given that its distribution at such small area level is likely to be homogenous. The strength of NTL data, however, is in their ease of extraction, their comparability across space and their repeated measurements.

Our findings on the relationship of asset indices and NTL in Africa are comparable with previous studies where various NTL metrics were shown to be useful indicators of economic activity and correlated with GDP [17] and income per-capita [15] in Europe and the USA. In combination with gridded global population maps, NTL brightness was also shown to be a relatively accurate metric for computing populations below national and international poverty lines [19]. However, there are issues of scale dependence [17,38] whereby different results can be observed from the same data aggregated at different geographic scales which can lead to erroneous imputations from observations at a smaller geographic unit to a larger one or vice versa [17]. In this analysis it is not clear whether the fidelity of our observations will remain when aggregated to resolutions finer than the Administrative 1 level in Africa. In addition, the NTL data used here suffer from a 'blooming' effect – the tendency to over-estimate the extent of large, well-electrified urban areas [18,39], a problem which the new generation of NTL products in production attempt to resolve [40]. It is possible, therefore, that the strength of the relationship between asset-based indices and NTL metrics observed at Administrative 1 level for Africa may not hold at lower resolution and caution should be exercised when extrapolating the findings of these results.

## Conclusion

The study shows that in Africa mean brightness of NTL is highly correlated with asset-based indices at the Administrative 1 level. The observations made here are plausible given that where there are more investments in infrastructural development, particularly in urban settings, people are on the whole wealthier [41]. The rate of urban development and electrification in Africa is discussed elsewhere [37]. What this study shows, however, is that public domain, spatially continuous and temporally dynamic data on NTL can be used to track changes in poverty levels and that these relate to current standards of poverty measurement.

## List of abbreviations

DMSP: Defense Meteorological Satellite Program; GDP: Gross Domestic Product; IMF: International Monetary Fund; MDGs: Millennium Development Goals; NOAA-NGDC: National Oceanic and Atmospheric Administration-National Geophysical Data Centre; NTL: Night-time lights; OLS: Operational Linescan System; PCA: Principal Component Analysis; SSA: sub-Saharan Africa; UN: United Nations; WHO: World Health Organization;

## Competing interests

The authors declare that they have no competing interests.

## Authors' contributions

AMN was responsible for study design, data cleaning, analysis, interpretation and production of the final manuscript. VAA contributed to data cleaning and analysis and production of final manuscript. PWG contributed to analysis, interpretation and production of the final manuscript. AJT contributed to analysis, interpretation and production of the final manuscript. RWS was responsible for overall scientific management, analysis, interpretation and preparation of the final manuscript.

## Funding source

AMN is supported by the Wellcome Trust as a Research Training Fellow (#081829). RWS is supported by the Wellcome Trust as Principal Research Fellow (#079081). Both AMN and RWS acknowledge the support of the Kenyan Medical Research Institute. This work forms part of the output of the Malaria Atlas Project (MAP: ), principally funded by the Wellcome Trust.

## References

[B1] World Bank (2001). Attacking poverty.

[B2] WHO (2001). Macroeconomics and Health: Investing in Health for Economic Development.

[B3] UN (2001). Road map towards the implementation of the United Nations millennium declaration.

[B4] International Monetary Fund Poverty Reduction Strategic Papers. http://www.imf.org/external/np/prsp/prsp.asp.

[B5] Deaton A, Zaidi S (2002). Guidelines for Constructing Consumption Aggregates for Welfare Analysis Living Standards Measurement Study Working Paper, No 135.

[B6] Hulme D, McKay A (2005). Identifying and Measuring Chronic Poverty: Beyond Monetary Measures.

[B7] Stifel D, Christieaensen L (2006). Tracking poverty over time in the Absence of Comparable Consumption Data World Bank Research Policy Working Paper 3810.

[B8] Filmer D, Pritchett LH (2001). Estimating wealth effects without expenditure data – or tears: an application to educational enrolments in states of India. Demography.

[B9] Demographic and Health Surveys http://www.measuredhs.com.

[B10] Multiple Indicator Cluster Surveys http://www.childinfo.org/mics.html.

[B11] Falkingham J, Namazie C (2002). Measuring health and poverty: A review of approaches to identifying the poor.

[B12] Sahn D, Stifel D (2000). Poverty comparisons over time and across countries in Africa.

[B13] Elbers C, Lanjouw JO, Lanjouw P (2001). Welfare in villages and towns: Micro-level estimation of poverty and inequality.

[B14] Lanjouw P (1998). Ecuador's rural nonfarm sector as a route out of poverty World Bank, Policy Research Working Paper No 1994.

[B15] Ebener S, Murray C, Tandon A, Elvidge C (2005). From wealth to health: modeling the distribution of income per capital at the sub-national level using nighttime light imager. Int J Health Geog.

[B16] Doll CNH, Muller JP, Elvidge CD (2002). Night-time Imagery as a Tool for Global Mapping of Socio-economic parameters and Greenhouse Gas Emissions. Ambio.

[B17] Doll CNH, Muller JP, Morley JG (2006). Mapping regional economic activity from night-time light satellite imagery. Ecol Econ.

[B18] Elvidge C, Baugh K, Kihn EA, Kroehl HW, Davis ER (1997). Mapping city lights with nighttime data from the DMSP operational linescan system. Photogramm Eng Rem Sens.

[B19] Elvidge CD, Sutton PS, Baugh KE, Tuttle BT, Howard AT, Erwin EH, Bhaduri B, Bright E A global poverty map derived from satellite data.

[B20] Pozzi F, Small C, Yetman G (2002). Modelling the distribution of human population with night-time lights imagery and gridded population of the world Pecora 15/Land Satellite Information IV/ISPRS Commission I/FIEOS Conference Proceedings.

[B21] Tobler W, Deichmann U, Gottsegen J, Maloy K (1995). The Global Demography Project, Technical Report TR-95-6.

[B22] Second Administrative Level Boundaries (SALB) http://www.who.int/whosis/database/gis/salb/salb_PO.htm.

[B23] Global Administrative Unit Layers (GAUL) http://www.fao.org/geonetwork/srv/cn/metadata.show?id=12691.

[B24] Guerra C, Gikandi P, Tatem A, Noor A, Smith D, Hay S, Snow R (2008). The limits and intensity of Plasmodium falciparum transmission: Implications for malaria control and elimination worldwide. PLoS Med.

[B25] Balk DL, Deichmann U, Yetman G, Pozzi F, Hay SI, Nelson A (2006). Determining global population distribution: methods, applications and data. Adv Parasitol.

[B26] Hay SI, Noor AM, Nelson A, Tatem AJ (2005). The accuracy of human population maps for public health application. Trop Med Int Health.

[B27] Tatem AJ, Noor AM, von Hagen C, di Gregorio A, Hay SI (2007). High resolution population maps for low income nations: combining land cover and census in East Africa. PLoS One.

[B28] Gridded Population of the World, version 3 (GPWv3) and the Global Rural-Urban Mapping Project (GRUMP). http://sedac.ciesin.columbia.edu/gpw/.

[B29] Cinzano P, Falchi F, Elvidge CD (2001). The first World Atlas of the artificial night sky brightness. Mon Not R Astron Soc.

[B30] Elvidge CD, Imhoff ML, Baugh KE, Hobson VR, Nelson I, Safran J, Dietz JB, Tuttle BT (2001). Night-time lights of the world: 1994–1995. J Photogramm Rem Sens.

[B31] DMSP Nighttime Lights of the World – Change Pair (Version 1).

[B32] Multiple Indicator Cluster Surveys http://www.childinfo.org/mics/mics3_surveys.html.

[B33] Viera AJ, Garrett JM (2005). Understanding interobserver agreement: The Kappa statistic. Fam Med.

[B34] UNDP Millennium Development Goals country reports 2007.

[B35] Space Physics Interactive Data Resource http://spidr.ngdc.noaa.gov/spidr/.

[B36] Elvidge CD, Cinzano, Pettit DR, Arvesen J, Sutton P, Small C, Nemani R, Longcore T, Rich C, Safran J, Weeks J, Ebener S (2007). The Nightsat mission concept. Int J Rem Sens.

[B37] Lighting Africa Catalysing markets for modern lighting.

[B38] Wrigley N, Holt T, Steel D, Tranmer M, Longley P, Batty M (1996). Analysing, modelling and resolving the ecological fallacy.

[B39] Tatem AJ, Noor AM, Hay SI (2005). Assessing the accuracy of satellite derived global and national urban maps in Kenya. Remote Sens Env.

[B40] Balk D, Pozzi F, Yetman G, Deichmann U, Nelson A (2004). The distribution of people and the dimension of place: Methodologies to improve global population estimates in urban and rural areas.

[B41] Fosto JC (2006). Child health inequities in developing countries: differences across urban and rural areas. Int J Equity Health.

